# A Quantum Biomimetic Electronic Nose Sensor

**DOI:** 10.1038/s41598-017-18346-2

**Published:** 2018-01-09

**Authors:** Ashlesha Patil, Dipankar Saha, Swaroop Ganguly

**Affiliations:** Indian Institute of Technology Bombay, Department of Electrical Engineering, Mumbai, 4000076 India

## Abstract

We propose a technologically feasible one-dimensional double barrier resonant tunneling diode (RTD) as electronic nose, inspired by the vibration theory of biological olfaction. The working principle is phonon-assisted inelastic electron tunneling spectroscopy (IETS), modeled here using the Non-Equilibrium Green Function formalism for quantum transport. While standard IETS requires low-temperature operation to obviate the thermal broadening of spectroscopic peaks, we show that quantum confinement in the well of the RTD provides electron energy filtering in this case and could thereby allow room-temperature operation. We also find that the IETS peaks - corresponding to adsorbed foreign molecules - shift monotonically along the bias voltage coordinate with their vibrational energy, promising a selective sensor.

## Introduction

The nascent field of quantum biology^1^ seeks to study biological systems where non-trivial quantum effects play functional roles. Examples of such systems are excitonic energy transfer in photosynthesis, avian magnetoreception, hydrogen tunneling in enzyme reactions, and, possibly, olfaction. Classical neurobiology holds that olfaction proceeds by a ‘lock and key’ mechanism whereby it is the structure of a molecule that is sensed^2^. There is, however, also a heterodox picture called the vibration theory of olfaction, which was proposed for the first time in 1938 by Malcolm Dyson and elaborated by R.H. Wright in 1977; it lay dormant until 1996, when Luca Turin gave it concrete shape by propounding inelastic quantum-mechanical tunneling as the mechanism for vibration sensing.

According to the vibration theory, the smell of a molecule is determined by its vibrational spectrum^3–5^. While some experimental evidence has emerged over the years in favor of a role for vibrational modes^6–10^, there is also evidence to the contrary that supports the classical picture^11–14^. An emerging theory, called the “swipe card” model combines the features of the classical and quantum theories^15,16^. As the name suggests, it proposes a ‘key’, but with information encoded on it. First, the odorant molecule (‘key’) must be structurally compatible with the corresponding receptor in order to bind (‘lock’); and second, that opens up an inelastic electron tunneling channel between receptor contacts corresponding to its specific vibrational energy, and is thereby smelled (sensed).

## Results and Discussion

### Theory

The vibration theory of olfaction owes its operating principle to the much older characterization technique called Inelastic Electron Tunneling Spectroscopy (IETS)^5^. The current-voltage (I-V) characteristic of a tunneling barrier, usually a metal-insulator-metal (MIM) structure^17^, exhibits a kink when the applied voltage supersedes the energy of a vibrational mode in the system, thereby opening up an inelastic tunneling channel; this kink is exhibited as a peak in the *d* ^2^ *I*/*dV* ^2^ characteristic, thus enabling spectroscopy of vibrational modes^18^. We note as an aside that the second derivative is practically measured by locking in on the second harmonic of a small signal in order to minimize the effect of noise^19^. IETS can be a powerful technique because of its sensitivity and its ability to detect modes that might be optically inaccessible^19^. However, IETS on an MIM tunnel barrier in the laboratory is practically limited to cryogenic temperatures because thermal broadening of the contact Fermi  levels^20^ leads to merging of spectroscopic peaks at higher temperatures^21^. On the other hand, if inelastic electron tunneling indeed plays a functional role in natural olfaction as some of the evidence now suggests, that would imply that nature has figured out a way around the thermal broadening problem. Even if natural olfaction is not IETS-based, a room-temperature IETS-based (viz. biomimetic, in the Turin sense) electronic nose is an intriguing possibility, with great promise for gas-sensing applications^22^ of importance such as environmental monitoring, healthcare and security. One way around the thermal broadening problem here might be energy-filtering through the use of quantum structures. In previous work, we have explored a one-dimensional (1D) molecular wire as a candidate for an IETS-based electronic nose^23^. However, we found that it would still be unable to resolve IET spectra at room temperatures due to thermal broadening. Patil^21^ has discussed using quantum dots (QDs) as electron energy filters to restrict electron energies at room temperature. As a QD has discrete energy levels, it allows only electrons with certain energies to flow. For sensing applications, we need arrays of QDs so as to get reasonable sensing area. But, a 2D array of QDs forms energy minibands unlike a single QD and hence fails to act as an efficient energy filter.

**Figure 1:**
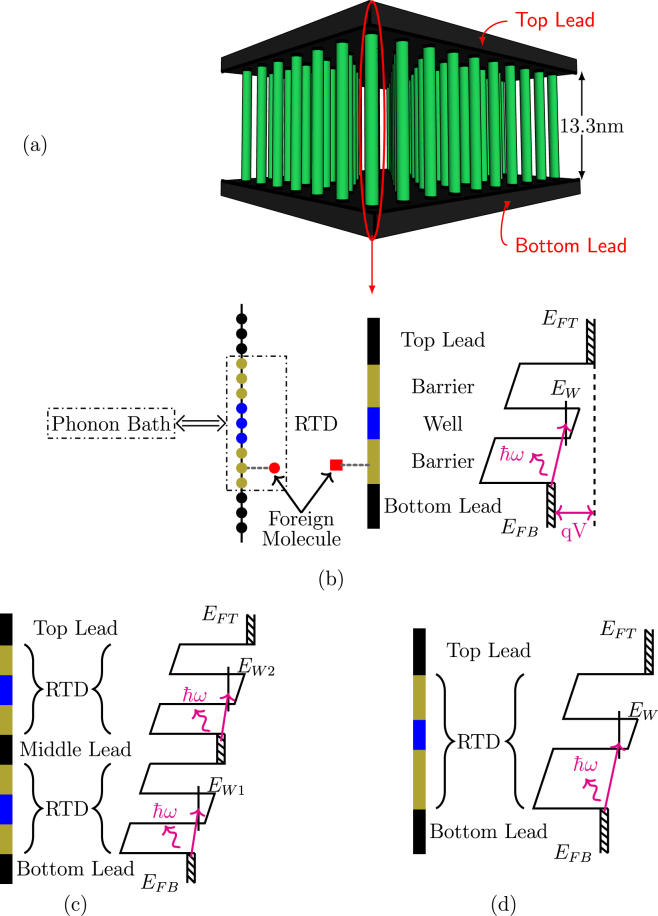
(**a**) 3D network of parallel nanowires. (**b**) From left to right - molecular arrangement, schematic structure, and band diagram (including illustration of phonon-assisted tunneling) for RTD nanowire. (**c**) From left to right - nanowire with symmetric RTDs connected in series, and corresponding band diagram. (**d**) From left to right - nanowire with asymmetric RTD, and corresponding band diagram.

In this work, we show using inelastic quantum transport simulations, that a 1D double-barrier resonant tunneling diode (RTD) can be used as an effective energy filter to achieve room temperature operation as an IETS-based electronic nose. Note that this work is focused on demonstrating the viability of inelastic tunneling as a sensing mechanism. Thus, we do not yet get into important sensor performance questions such as quantitative estimation of sensitivity, surface functionalization etc. Even so, we propose a 3D network of such RTDs connected in parallel to common leads as shown in Fig. 1a. This type of arrangement obviously helps to increase the sensor area. Figure 1b shows the schematic of a 1D (nanowire) symmetric RTD connected to semi-infinite leads and its conduction band with phonon-assisted inelastic tunneling. Before taking up its working principle, we point out that if a RTD gets shorted out for some reason, that would affect the total current flowing through the network and lead to an erroneous vibration spectrum; therefore, we propose two or more RTDs connected in series in each nanowire as shown in Fig. 1c. We note that the device structure is within the reach of current technology^24^, requiring superlattice nanowire growth^25–27^ to be wedded to vertical nanowire device fabrication^28–33^.

Now, for a single RTD, it is well-known that when the applied voltage is such that the resonant (broadened, quasi-stationary state) energy level in the well is pulled down below the conduction band edge on the emitter side, electrons cannot tunnel through the barrier elastically, and the resonant current drops precipitously to give a well-known negative differential resistance region^34^. However, an electron could lose energy by emitting a vibron (quantum of localized molecular vibration) or phonon (quantum of collective vibration of the material), and thereby open up an additional tunneling channel at an applied voltage for which *E*_*FB*_ − *E*_*W*_ = *ħω*, where *ħω* is the phonon/vibron mode energy. In this case, electrons can tunnel inelastically to the energy level in the well. This process manifests itself in a second, typically smaller, satellite peak in the I-V characteristics. The presence of the satellite peak has been experimentally demonstrated by Goldman *et al*.^35^. The peaks in the IET spectrum corresponding to the satellite peak in I-V characteristics can then be used as the signature of vibrational modes in this case. We find that the position of the IETS peak maps monotonically to the vibrational mode energy present in the device and thereby identify an adsorbed odorant molecule.

In our simulation, we assume that electron-phonon interaction is present only in the RTD region and not in contacts. We employ the Non-Equilibrium Green’s Function (NEGF) formalism within the self-consistent Born approximation to describe inelastic tunneling transport which is discussed in detail in the Appendix. We have chosen the following set of parameters for simulations: The RTD is assumed to be constituted of barriers comprising fifteen atomic sites each and a well region comprising ten sites at temperature *T* = 300 K with *ε* = 0 eV, *t *= 5.2 eV, [See Fig. 1b for reference]. The contact Fermi energy *E*_*FB*_ = 20*meV* and *E*_*FB*_ − *E*_*FT*_ = *V*_*A*_, where *V*_*A*_ is the applied bias and the barrier height *V*_*B*_ = 0.6 *eV*. The electron-phonon (e-ph) coupling energy is same at each grid point for a given phonon mode.

### Observations

**Figure 2:**
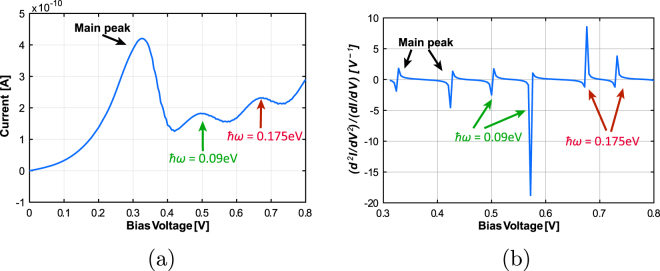
Current-voltage characteristics and IET spectrum for nanowire RTD with two bulk phonon modes (*ħω*_1_ = 0.09 eV and *ħω*_2_ = 0.175 eV).

We start with a simple demonstration of the simulation setup, looking at quantum transport across the RTD with collective phonon modes present in the system. Figure 2 shows the simulated I-V characteristics and IET spectrum of the RTD with two phonon modes with energies *ħω*_1_ = 0.09 eV and *ħω*_2_ = 0.175 eV. We observe from Fig. 2a that, in addition to the main peak, there are two satellite peaks in the I-V characteristics due to the two phonon modes; Fig. 2b shows the corresponding IET spectrum. Each satellite peak in the I-V results in a set of two peaks in the IETS, one for the local maximum and another for the local minimum. Thus, the peaks at *V* = 0.5 V and *V* = 0.68 V correspond to the local maxima for *ħω*_1_ = 0.09 eV and *ħω*_2_ = 0.175 eV respectively. The first two peaks represent the peak and valley current of the RTD. As the I-V is not smooth, the second derivative is quite noisy. To mitigate this, we apply a simple second order Butterworth filter to smoothen the IET spectrum here; more sophisticated circuitry may be used for this purpose in actual hardware. With that, we are in principle able to identify the bulk phonon modes present in the RTD using IETS at room temperature. Now, the presence of discernible satellite peaks in the I-V might raise a question about the need to take the second derivative, viz. IETS. However, the peaks here are perceptible because the phonon mode used is a bulk mode, i.e. it is present everywhere in the device. But if the mode is localized, the satellite peak itself might be too weak to be discernible. In this case, which could be the default for sensing applications, it then becomes necessary to get the IET spectrum.

**Figure 3:**
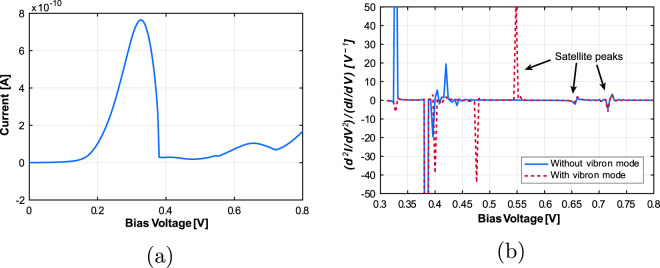
Current-voltage characteristics and IET spectrum for nanowire RTD with an odorant molecule attached to the emitter side barrier (bulk phonon mode energy *ħω* = 0.175 eV and vibron energy *ħω*_*f*_ = 0.09 eV).

In order to investigate the basic sensing functionality, we model the effect of a foreign molecule attached to a particular site in the device as a modulation of the hopping term t at that site. In our case, it is modified to *t*_*p*_ = 4.5 eV. In Fig. 3, the localized phonon mode associated with the foreign molecule has energy *ħω*_*f*_ = 0.09 eV and the bulk phonon mode has *ħω* = 0.175 eV. The foreign molecule is attached to a site in the emitter side (bottom) barrier as shown in Fig. 1b. Here the satellite peak in the I-V characteristics (Fig. 3a) due to the localized mode is not visible and we need to calculate the IET spectrum to detect it. In Fig. 3b, the IETS peak for the localized mode can be seen at *V *= 0.55 V along with the peak at *V *= 0.65 V corresponding to the bulk mode. Here we have not applied any smoothing filter to the IET spectrum as that would also flatten the weak peaks due to localized mode.

**Figure 4:**
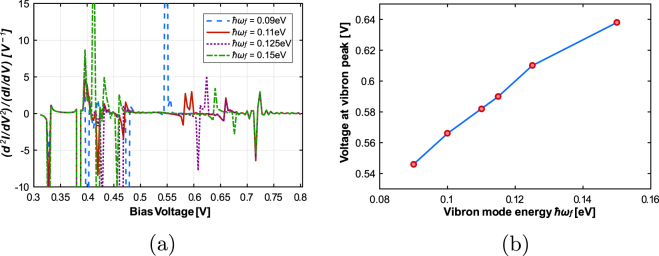
Nanowire RTD IET spectrum, and, IETS peak position (in voltage) vs vibron energy for different values of vibron energy and a fixed bulk phonon mode energy (*ħω* = 0.175 eV).

Next, we take a look at how this device would sense various types of odorants, distinguished by their vibrational energies. Figure 4a shows the IET spectrum for the RTD with a bulk phonon mode *ħω *= 0.175 eV and vibron with energy ranging from 0.09 eV to 0.15 eV placed in the bottom barrier layer. The peaks beyond 0.5 V correspond to the phonon peaks and those before 0.5 V are due to the peak and valley current of the RTD. We see that the position of the peak due to the localized mode shifts uniformly with the vibrational energy (Fig. 4b). This demonstrates that we can distinguish between odorant molecules with different phonon mode energies using the RTD. We recognize that the sensor will not be able to differentiate between non-identical molecules with equal vibrational energies; but this may not be very different from biological olfaction where molecules with very different structure but similar vibrational energy have been found to smell similar^5^. We also note that if the peaks for the bulk and localized modes are very close to each other, the bulk mode will dominate over the localized mode. Hence, we are able to identify only those odorant molecules whose vibrational energies lie in a particular range.

**Figure 5:**
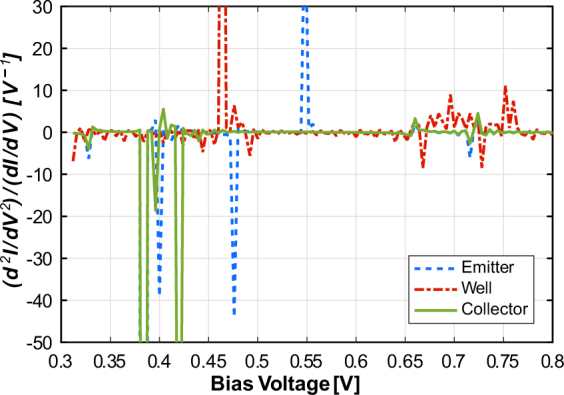
IET spectrum for placement of odorant molecule at different regions of nanowire RTD (bulk phonon mode energy *ħω* = 0.175 eV and vibron energy *ħω*_*f*_ = 0.09 eV).

A practical consideration in device design here is the available area for adsorbing foreign molecules. In particular, for this device, the question is whether all its regions - well and barriers - are equally effective for this purpose. Figure 5 shows the IET spectrum for a foreign molecule located in different regions of the RTD. We observe that the IETS peak corresponding to the localized mode is absent if the foreign molecule is attached anywhere except the emitter side (bottom) barrier. We can infer that the incoming electron excites vibrational modes predominantly in the barrier region so as to tunnel across when the energy level *E*_*W*_ in the well falls bellow the conduction band in the emitter. This renders the rest of the RTD ineffective for sensing the foreign molecule, and thus, the effective sensing region is only the emitter side barrier. In order to increase the sensing area of a single RTD, we, therefore, propose using an asymmetric RTD with wider emitter side barrier as shown in Fig. 1d.

### Conclusion

We have investigated the double barrier resonant tunneling device as a potential candidate to overcome the thermal broadening problem in IETS using quantum confinement for energy filtering. We have demonstrated theoretically that it is possible to detect foreign odorant molecules at room temperature using this structure and thereby established its feasibility as an electronic nose. We have observed that only one of the barriers of the RTD, that on the emitter side, is effective for detection of foreign molecules; this suggests that asymmetric device designs ought to be explored. The RTD design considered in this work can detect molecules with vibrational energy roughly from 0.09 eV to 0.15 eV. Explosives such as Nitramine (RDX), TNT and Nitrate ester (PETN) have prominent signatures in this range of the vibration spectrum^36–38^. For a given compound detection, we can modify the height and width of the RTD to change the energy range of detection and use multiple RTDs in conjunction to measure the entire vibration spectrum. We have proposed a 3D parallel nanowire architecture for improving the total sensing area, with two RTDs in series to afford short protection. The proposed sensing device brings us a step closer to the emulation of the vibration model of animal olfaction and raises the hope of realizing an electronic nose that can truly rival natural ones. Further work needs to focus on the fabrication of such quantum devices, their surface functionalization, and importantly, optimized signal processing circuitry for the second derivative extraction. Thereafter, one would be in a position to realistically quantify the sensitivity and selectivity of the sensor and benchmark it to existing systems.

## Methods

The NEGF modeling for a 1D RTD is very similar to that of a quantum wire using a 1D grid and can be found in^39^. We model the RTD region with *N* sites connected to semi-infinite leads using the effective mass Hamiltonian *H*_*RTD*_ obtained by using the finite difference method on atomistic Hamiltonian^40^. Here onsite energy is *ε *+ 2*t* and the hopping energy is −*t*.

1 $${H}_{RTD}={[\begin{array}{cccc}\varepsilon +2t & -t &  & 0\\ -t & \varepsilon +2t & -t & \\  & \ddots  & \ddots  & -t\\ 0 &  & -t & \varepsilon +2t\end{array}]}_{N\times N}$$

We calculate the retarded Green’s Function using:

2 $$G(E)={[(E+\iota {\eta }^{+})I-{H}_{RTD}-{{\rm{\Sigma }}}_{B}(E)-{{\rm{\Sigma }}}_{T}(E)-{{\rm{\Sigma }}}_{scat}(E)]}^{-1}$$

here, Σ_*B*_(*E*), Σ_*T*_(*E*) and Σ_*scat*_(*E*) are the self energies corresponding to the bottom lead, the top lead and the phonon bath respectively [refer Fig. 1b]. The contact self energies, Σ_*B*_(*E*) and Σ_*T*_(*E*) incorporate contact boundary conditions in the Hamiltonian. Similarly, Σ_*scat*_(*E*) includes the effects of phonon bath which can be treated as a scattering terminal akin to the “Büttiker probe”. Hence, the total current flowing through this terminal is equal to zero.

3 $${{\rm{\Sigma }}}_{scat}(E)=\frac{-\iota }{2}[{{\rm{\Sigma }}}_{scat}^{in}(E)+{{\rm{\Sigma }}}_{scat}^{out}(E)]$$

The electron(hole) correlation functions are given by:

4 $${G}^{n(p)}(E)=G{{\rm{\Sigma }}}^{in(out)}{G}^{\dagger }$$

where $${{\rm{\Sigma }}}^{in(out)}={{\rm{\Sigma }}}_{B}^{in(out)}+{{\rm{\Sigma }}}_{T}^{in(out)}+{{\rm{\Sigma }}}_{scat}^{in(out)}$$.

The in/out-scattering functions for phonons ${{\rm{\Sigma }}}_{scat}^{in(out)}$ at a particular site can be calculated within self-consistent Born approximation using the following equations:

5 $${{\rm{\Sigma }}}_{sca{t}_{q}}^{in}(E)=\sum _{{\eta }}{D}_{q}^{\eta }[n(\hslash \omega ){G}^{n}(E-\hslash \omega )+(n(\hslash \omega )+\mathrm{1)}{G}^{n}(E+\hslash \omega )]$$

6 $${{\rm{\Sigma }}}_{sca{t}_{q}}^{out}(E)=\sum _{{\eta }}{D}_{q}^{\eta }[n(\hslash \omega ){G}^{p}(E+\hslash \omega )+(n(\hslash \omega )+\mathrm{1)}{G}^{p}(E-\hslash \omega )]$$

where ${D}_{q}^{\eta }$ is e-ph coupling constant at grid point q for phonon mode *η* with energy *ħω*. *n*(*ħω*) is Bose distribution function for phonons given by $1/\{{\exp }[\hslash \omega /({k}_{B}T)]+1\}$. The total current through the RTD is given by:

7 $$I=\frac{2e}{h}\int Tr[{{\rm{\Sigma }}}_{B}^{out}(E){G}^{n}(E)-{{\rm{\Sigma }}}_{B}^{in}(E){G}^{p}(E)]dE$$

**Figure 6:**
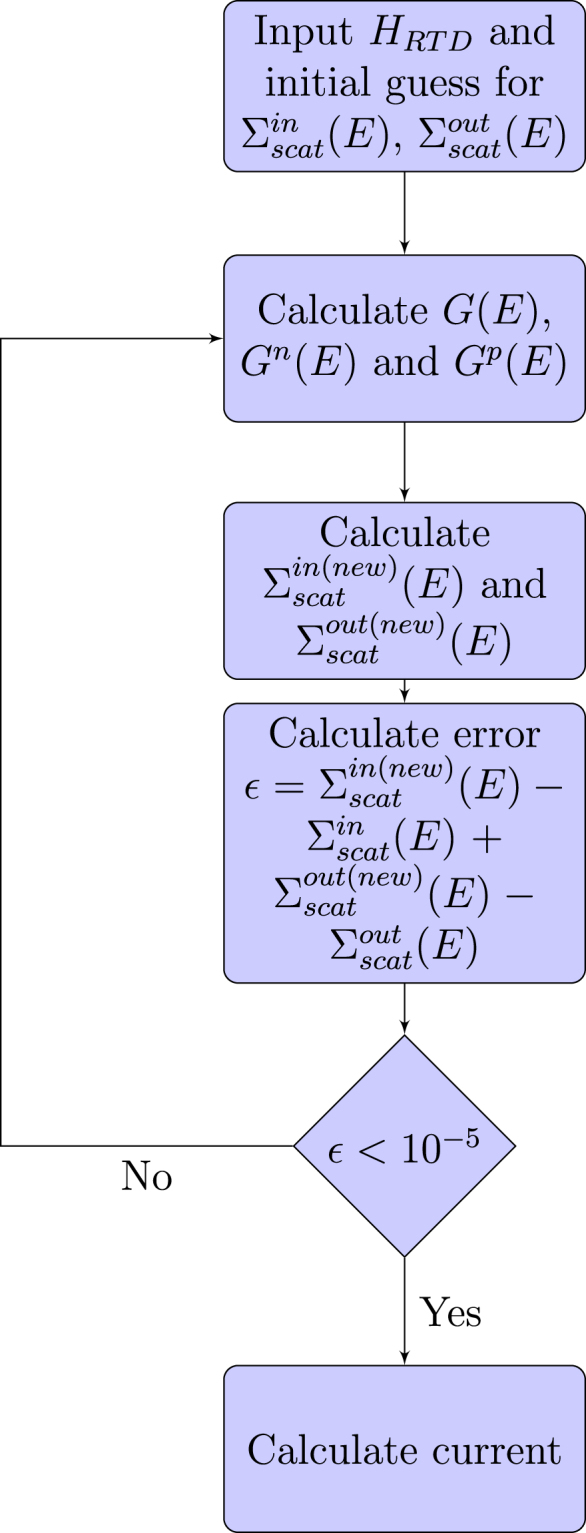
Algorithm for calculating the current using the NEGF method within the self-consistent Born approximation.

From (4), (5) and (6), it can be seen that *G* ^*n*(*p*)^(*E*) and ${{\rm{\Sigma }}}_{scat}^{in(out)}$ are interdependent and hence need to be solved self-consistently. The algorithm to calculate current is shown in Fig. 6. We then take the second derivative of the current with respect to the applied voltage in order to calculate its IET spectrum.
